# Osteoprotegerina e Disfunção Vascular em Pacientes com Doença Renal Crônica Estágio 3 e em Pacientes sem Disfunção Renal: Estudo Caso-Controle

**DOI:** 10.36660/abc.20240122

**Published:** 2024-11-07

**Authors:** Thalita de Oliveira Matos, Priscila Valverde de Oliveira Vitorino, Rogerio Orlow, Átila de Oliveira Melo, Diogo da Silva Amorim, Gleidson Junio Oliveira Sousa, Vanda Jorgetti, Ana Luiza Lima Sousa, Rodrigo Bezerra, Weimar Kunz Sebba Barroso

**Affiliations:** 1 Universidade Federal de Goiás Goiânia GO Brasil Universidade Federal de Goiás – Liga de Hipertensão, Goiânia, GO – Brasil; 2 Pontifícia Universidade Católica de Goiás Escola de Ciências Sociais e da Saúde Goiânia GO Brasil Pontifícia Universidade Católica de Goiás – Escola de Ciências Sociais e da Saúde, Goiânia, GO – Brasil; 3 Universidade Federal de Goiás Goiânia GO Brasil Universidade Federal de Goiás, Goiânia, GO – Brasil; 4 Universidade de São Paulo São Paulo SP Brasil Universidade de São Paulo, São Paulo, SP – Brasil; 5 Universidade de Pernambuco Recife PE Brasil Universidade de Pernambuco, Recife, PE – Brasil

**Keywords:** Rigidez Vascular, Calcificação Vascular, Osteoprotegerina, Endotélio Vascular, Doenças Cardiovasculares, Insuficiência Renal Crônica

## Abstract

**Fundamento:**

A osteoprotegerina (OPG) é um marcador de calcificação vascular e de risco cardiovascular em pacientes com doença renal crônica (DRC).

**Objetivos:**

Comparar e correlacionar os valores de OPG com as medidas de dilatação fluxo-mediada (DFM) e de velocidade de onda de pulso (VOP) em pacientes com DRC estágio 3 e em indivíduos sem disfunção renal.

**Métodos:**

Este estudo caso-controle foi conduzido em um centro especializado em hipertensão em 2022. Um total de 79 pacientes com idade acima de 18 anos participou do estudo. O grupo caso foi composto de 30 pacientes com disfunção renal moderada (DRC estágio 3) e o grupo controle incluiu 49 indivíduos com uma taxa de filtração ≥ 60 mL/min/1,73 m^2^. O nível de significância adotado na análise estatística foi de 5%.

**Resultados:**

A pressão de pulso central (PPC), a VOP e o índice de aumento (IA) foram mais altos nos pacientes com disfunção renal. Os níveis séricos de OPG mostraram uma correlação positiva com a pressão arterial sistólica central e periférica, PPC, VOP e IA. Por outro lado, valores séricos de OPG não se correlacionaram com DFM.

**Conclusões:**

A OPG e a VOP são possíveis biomarcadores de disfunção vascular que se encontram alterados em pacientes com disfunção renal moderada. Apesar de limitações deste estudo, incluindo o fato de ser um estudo caso-controle conduzido em um único centro, o estudo tem o potencial, como uma prova de conceito, de gerar a hipótese de que tanto a OPG como a VOP são biomarcadores de dano vascular precoce nessa população.

## Graphical abstract

**Figura Central:**
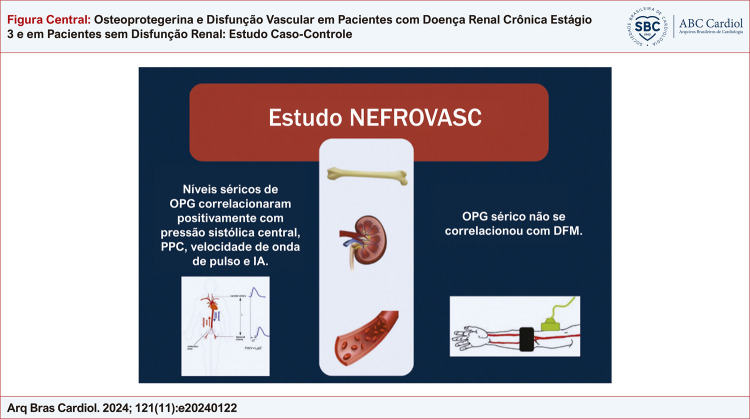
: Osteoprotegerina e Disfunção Vascular em Pacientes com Doença Renal Crônica Estágio 3 e em Pacientes sem Disfunção Renal: Estudo Caso-Controle

Osteoprotegerina e outras variáveis: principais achados; IA: índice de aumento; DFM: dilatação fluxo-mediada; OPG: osteoprotegerina; VOP: velocidade de onda de pulso; PPC: pressão de pulso central.

## Introdução

Pacientes com doença renal crônica (DRC) têm risco aumentado de desenvolverem doença cardiovascular (DCV) devido a fatores de risco (FR) tradicionais como diabetes mellitus (DM), hipertensão (HT), dislipidemia (DLP), tabagismo, e idade. Esse risco é ainda maior nesses pacientes devido a FR não tradicionais, como distúrbios do metabolismo mineral e ósseo, inflamação crônica, anemia, aumento de toxinas urêmicas, depleção de óxido nítrico endotelial, acúmulo de produtos finais da glicosilação, ativação aumentada do sistema renina-angiotensina-aldosterona (SRAA), e estresse oxidativo. Esses FR potencializam a disfunção endotelial, calcificação vascular e osteogênese observados nos pacientes com DRC.^1,2^

A disfunção endotelial está associada ao desenvolvimento de DCV, e sua avaliação é realizada pelo método de dilatação fluxo-mediada (DFM). Embora a DFM seja o método considerado padrão-ouro,^3,4^ resultados de estudos com pacientes com DRC continuam controversos, principalmente naqueles com estágios menos avançados da doença.^5-7^

Outro método utilizado para identificar lesão vascular precoce são as medidas da pressão arterial central (PAC) e da velocidade de onda de pulso (VOP). Ambos os parâmetros são bons desfechos substitutos^8^ e preditores independentes de mortalidade por DCV em indivíduos com DRC.^9-11^ Cada 1m/segundo de elevação na VOP corresponde a um aumento de 15-30% no risco de morte cardiovascular (CV).^12^ Ainda, um aumento na pressão de pulso central (PPC) a valores superiores a 50mmHg está associado a uma piora na função renal e nos desfechos CVs adversos.^12-14^

A calcificação vascular é comumente encontrada em pacientes com DRC avançada.^15^ Esses pacientes apresentam alterações fenotípicas que culminam em distúrbios homeostáticos de cálcio, fósforo, paratormônio, e calcitriol, além de outros distúrbios relacionados ao metabolismo e ao remodelamento ósseo, com reabsorção aumentada e calcificação heterotrópica, principalmente calcificação vascular.^16,17^

A calcificação vascular é um processo ativo no qual existem alterações na composição da matriz do vaso e mudanças fenotípicas de células musculares lisas a células similares a osteoblastos. Ainda, há a presença de proteínas moduladoras de osteogênese e de um desequilíbrio entre fatores estimuladores e inibitórios da calcificação vascular.^17-19^

A Osteoprotegerina (OPG) é uma glicoproteína da família do Fator de Necrose Tumoral (TNF) que previne a ligação do RANK-L ao Receptor Ativador de Fator Nuclear kappa-B (RANK), inibindo, assim, a ativação de osteoclasto. Assim, acredita-se que a OPG iniba a osteoclastogênese e a calcificação, e proteja o volume ósseo e a massa óssea.^20^ Níveis circulantes de OPG estão elevados em pacientes que necessitam de diálise e podem ser um forte preditor de calcificação vascular em vários estágios da doença renal.^21-23^

A lesão vascular é possivelmente um dos determinantes de alto risco CV em pacientes com DRC e a detecção desse dano em estágios iniciais da doença pode contribuir para a adoção de estratégias com o potencial de reduzir a morbidade e a mortalidade CV e renal. Este estudo teve como objetivo verificar a correlação entre OPG sérica e diferentes graus de disfunção vascular em pacientes com DRC estágios 3a e 3b e a comparar com pacientes sem DR.

## Métodos

Este é um estudo caso-controle que incluiu pacientes de um centro de referência em HT – a Liga de Hipertensão Arterial da Universidade Federal de Goiás. O estudo incluiu 79 participantes com um ou mais FR CV. Os participantes foram classificados de acordo com sua função renal, determinada por taxa de filtração glomerular (TFG) estimada pela equação da Colaboração em Epidemiologia da DRC (2001). Neste estudo, pacientes com uma TFG < 60mL/m^2^ e ≥30 mL/min/1,73 m^2^foram classificados como pacientes com DRC 3a e 3b, respectivamente. Indivíduos com DRC estágio 3a apresentam uma TFG entre 45 e 59, e aqueles com DRC estágio 3b apresentam uma TFG entre 30 e 44 mL/min/1.73 m^2^. Pacientes com uma TFG ≥60 mL/min/1,73m^2^ foram classificados como pacientes sem DR. O estudo foi aprovado pelo comitê de ética do Hospital das Clínicas da Universidade Federal de Goiás (UFG) (CAAE: 47327421.7.0000.5078).

Foram incluídos adultos com idade igual ou superior a 18 anos, com HT e uma TFG≥30 mL/min/1,73m^2^durante acompanhamento regular em um centro de referência de HT. Os critérios de exclusão foram pacientes com DRC que necessitavam de hemodiálise ou de diálise peritoneal, pacientes com transplante renal prévio, gestantes ou mulheres no pós-parto, pacientes com diagnóstico de câncer, pacientes em quimioterapia ou radioterapia, pacientes com história de doença sistêmica autoimune, pacientes em uso de imunossupressores, pacientes com hepatopatia crônica avançada (Child B e C), e aqueles com DCV prévia.

DCV prévia foi definida como presença de qualquer um dos seguintes diagnósticos: doença coronariana (angina, infarto agudo do miocárdio), insuficiência cardíaca, doença valvar cardíaca, arritmias complexas, doença arterial obstrutiva periférica, aneurisma aórtico, acidente vascular cerebral, e doença aterosclerótica caracterizada por estenose crítica no leito vascular. Os diagnósticos de HT, DM e DLP foram feitos com base na história clínica registrada nos prontuários médicos e pelo uso crônico e regular de medicamentos para o tratamento dessas doenças.

Os grupos de pacientes com ou sem disfunção renal (DR) foram definidos segundo testes realizados em um único laboratório. Todos os pacientes com DR foram recrutados do centro de referência e um grupo controle também foi formado com pacientes sem DR, pareados quanto a presença de DM e obesidade.

Os participantes foram submetidos a exames físicos e laboratoriais. A pressão arterial periférica foi medida de acordo com a técnica recomendada pelas Diretrizes Brasileiras de Hipertensão,^24^ utilizando-se um aparelho oscilométrico automático (Omron HBP-1100). Foram tomadas três medidas consecutivas com o paciente sentado, e a média das duas últimas medidas foi registrada como a pressão arterial periférica. Na mesma consulta, foi coletada amostra de sangue para exames laboratoriais, e foram avaliadas a PAC e DFM (Tabela 1).

**Tabela 1:** – Exames usados no estudo para avaliação de fatores de risco para disfunção vascular

Teste	Fator de risco avaliado
Osteoprotegerina	Calcificação vascular
Dilatação fluxo-mediada, albuminúria	Função endotelial
Medida da pressão arterial central	Rigidez vascular

A medida da PAC foi realizada usando a abordagem padronizada com o aparelho Dyna-MAPA-AOP (Cardios, Brasil) pelo método oscilométrico. O método foi aplicado segundo as recomendações da *American Heart Association*, 2015.8 O valor de referência para VOP para lesão de órgão-alvo foi de ≥10 m/s.

Os exames laboratoriais incluíram valores em jejum de glicemia, hemoglobina glicada, perfil lipídico (colesterol total e frações), creatinina, ureia e albuminúria. Foi coletada amostra (aproximadamente 30mL) de sangue venoso da veia antecubital entre 7am e 9am. Todos os participantes foram solicitados a fazer jejum por 12 horas. Também foi coletada uma amostra de 50mL de urina (jato médio) para análise de albuminúria.

Uma amostra de 15mL de sangue foi também coletada para avaliação laboratorial de OPG. Cada amostra foi centrifugada e o sobrenadante dividido em dois frascos (Eppendorf de 2mL) que foram rotulados e armazenados a -30^o^C. Amostras hemolisadas ou lipêmicas foram descartadas, e novas amostras foram colhidas. Foram evitados ciclos de congelamento e descongelamento, que poderiam causar resultados errôneos.

O nível sérico de OPG foi determinado usando a técnica ELISA sanduíche (BioVendor, República Tcheca) seguindo as instruções do fabricante. O teste usa um anticorpo monoclonal anti-OPG aderido à placa para capturar OPG no soro. A OPG capturada foi detectada pela adição de um segundo anticorpo policlonal anti-OPG conjugado com biotina.

A avalição da função endotelial por DFM foi realizada por um mesmo examinador treinado usando a técnica descrita por Celermajer et al.com um aparelho de ultrassom de alta resolução.^25^ O aparelho utilizado foi o UNEX EF38G, que possui um dispositivo robótico que escaneia a artéria braquial, fornecendo uma avaliação mais precisa. Atualmente, esse é o método padrão-ouro. O exame foi realizado inflando-se o manguito no antebraço a uma pressão de 50 mmHg acima da pressão arterial sistólica, e mantido inflado por cinco minutos. O pico do diâmetro da artéria braquial foi medido por avaliação contínua, dinâmica por ultrassonografia, até 180 segundos após a deflação do manguito.^4^ O risco de DCV é maior quando o DFM for inferior a 10%. Neste estudo, valores acima de 10% foram considerados normais.^26^

Para realizar s medida de PAC e a DFM, os pacientes foram orientados a passarem uma boa noite de sono na noite anterior ao exame, evitar bebidas contendo cafeína 12 horas antes, evitar bebidas alcóolicas 12 horas antes, não fumar seis horas antes, evitar exercícios físicos nas últimas 24 horas antes do exame, descansar por 20 minutos antes do exame, continuar o uso de medicamentos já utilizados, e fazer jejum de 12 horas.^4,26^ A Tabela 1 resume os exames realizados e os FR avaliados no estudo.

### Análise estatística

Um formulário eletrônico foi construído usando o REDCap^27^ e extraído com o JAMOVI, versão 2.2 para análise estatística.^28^ Análise descritiva foi expressa com frequência absoluta e relativa para variáveis qualitativas, e com média e desvio padrão ou mediana e intervalo interquartil para variáveis quantitativas, de acordo com a normalidade dos dados. O nível de significância adotado foi de 5%. Um cálculo amostral foi realizado para constituir a amostra.

Para uma diferença média de 4,4 2.1 pmo1/L em OPG entre pacientes no primeiro (4,9 ± 1,9 pmol/L) e no quarto quartil (9,3 ± 4,0 pmol/L), uma amostra de 19 participantes foi obtida em cada grupo. Dado o risco de se perder amostra coletadas para análise de OPG ou dificuldades em realizar DFM em um paciente, escolhemos aumentar nosso tamanho amostral para assegurar uma amostra significativa.

Para a análise comparativa entre os pacientes com e sem DR, o teste do qui-quadrado foi usado para as variáveis qualitativas e o teste t independente ou o teste de Mann-Whitney para as variáveis quantitativas. A análise comparativa da OPG de acordo com a classificação segundo as diretrizes KDIGO (*Kidney Disease: Improving Global Outcomes*) foi realizada usando o teste de Kruskal-Wallis com o teste post-hoc de Dunn entre os pacientes com diferentes estágios de DRC. Correlações da OPG com as outras variáveis foram analisadas usando os testes de correlação de Pearson e de Spearman.

## Resultados

A idade média dos participantes foi 64,1±9,7 anos. Os pacientes com DR eram mais velhos 70,1±7,2 vs. 60,0±8,7 anos (p <0,001) e apresentaram valores mais baixos de TFG. A distribuição de sexo foi similar entre os grupos (Tabela 2). O grupo com DR foi composto de 30 paciente e o grupo sem DR foi composto de 49 pacientes.

**Tabela 2:** – Descrição da amostra e comparação entre os pacientes com e sem disfunção renal

	Total	Com disfunção	Sem disfunção	p
Idade (anos)	64,1 ± 9,7	70,1 ± 7,2	60,0 ± 8,7	**< 0,001**
Índice de massa corporal (Kg/m^2^)	29,7 ± 5,9	28,9 ± 5,13	30,3 ± 6,38	0,301
TFG (ml/min/1,73 m^2^)	70,0 (50,5-88,0)	47,0 (42,3-53,0)	85,0 (72,0 – 95,0)	**<0,001**
≥ 65 anos	40 (50,6%)	22 (73,3%)	18 (36,7%)	**0,002**
Sexo feminino	47,0 (59,5 %)	18 (60,0%)	29 (59,2%)	1,000
Diabetes mellitus	27 (34,2%)	12 (40,0%)	15 (30,6%)	0,542
Dislipidemia	60 (78,9%)	23 (76,7%)	37 (80,4%)	0,916

Teste t independente ou teste de Mann-Whitney.

O uso combinado de anti-hipertensivos foi evidenciado em 82,3% da amostra. Inibidores da Enzima Conversora de Angiotensina (IECA) e Bloqueadores de Receptor de Angiotensina (BRA) foram os mais utilizados. Dos pacientes em uso de diuréticos, nenhum usava diuréticos de alça. Os diuréticos utilizados foram tiazida, diuréticos semelhantes à tiazida, e diuréticos poupadores de potássio (Tabela 3).

**Tabela 3:** – Frequência, número de drogas anti-hipertensivas, e classes de anti-hipertensivos usados por todos os participantes do estudo, e por pacientes com e sem disfunção renal

	Total (79 pacientes) n (%)	Com DR (30 pacientes) n (%)	Sem DR (49 pacientes) n (%)
**Número de anti-hipertensivos**			
Um	14 (17,7)	02 (6,6)	12 (24,4)
Dois	24 (30,4)	10 (33,3)	14 (28,5)
Três	24 (30,4)	12 (40,0)	12 (24,4)
Mais de três	17 (21,5)	06 (20,0)	11 (22,4)
**Classes de anti-hipertensivos**			
IECA/BRA	74 (93,7)	28 (93,3)	46 (93,8)
Diuréticos	55 (69,6)	20 (66,6)	35 (73,4)
BCC	42 (53,2)	20 (66,6)	22 (44,9)
Vasodilatadores	06 (7,6)	02 (6,6)	04 (66,7)
Betabloqueadores	21 (26,6)	10 (33,3)	11 (52,4)

BRA: bloqueador de receptor de angiotensina; IECA: inibidores da enzima conversora de angiotensina; BCC: bloqueadores de canais de cálcio; DR: disfunção renal.

Os valores de pressão arterial sistólica e diastólica, determinados por medidas periféricas e centrais, foram similares entre os grupos. Não foram observadas diferenças de valore de DFM entre os grupos. Ainda, PAC, VOP e índice de aumento (IA) foram mais altos no grupo de pacientes com DR (Tabela 4).

**Tabela 4:** – Comparação de resultados bioquímicos, pressão arterial periférica, medida de Pressão Arterial Central (PAC), e dilatação fluxo mediada entre pacientes com e sem disfunção renal

Exames	Total	Com DR	Sem DR	p
PAS periférica (mmHg)	137,0 (126,3–59,0)	146,3 (128,6–62,8)	135,5 (126,0–55,0)	0,208
PAD periférica (mmHg)	81,8±14,8	79,0±11,1	82,2±15,9	0,473
PAS central (mmHg)	112,0 (103,0-127,5)	112,5 (105,2-136,0)	112,0 (103,0-127,0)	0,410
PAD central	79,0 (70,0 – 91,5)	79,5 (69,3 – 90,3)	78,0 (71,0 – 93,0)	0,743
PPC (mmHg)	34,0 (29,0 – 40,0)	38,5 (32,0-47,0)	33,0 (27,5 – 37,0)	**0,006**
PWV (m/sec)	9,0 (8,6 – 10,2)	10,2 (9,2 – 11,9)	8,5 (7,7 – 9,5)	**<0,001**
IA (%)	24,0 (16,0 – 31,5)	27,0 (21,1 – 34,0)	21,0 (14,5 – 29,0)	**0,012**
DFM (%)	8,7 (5,3 – 12,4)	9,2 (5,0 – 11,4)	8,4 (5,4 – 13,4)	0,671
Creatinina(mg/dL)	1,1 (0,9 – 1,3)	1,4 (1,2 – 1,6)	0,9 (0,8 – 1,0)	**<0,001**
Ureia (mg/dL)	37,0 (29,0 – 45,0)	46,6 (39,0 – 58,8)	32,0 (25,5 – 71,5)	**<0,001**
Albuminúria (mg/g)	12,0 (5,0 – 28,2)	9,2 (2,0 – 22,8)	17,5 (7,6 – 61,4)	0,065
Glicemia (mg/dL)	95,0 (87,5 – 105,5)	95,0 (86,0 – 100,0)	97,0 (92,0 – 109,0)	0,168
HG (%)	5,7 (5,4 – 6,05)	5,8 (5,5 – 6,3)	5,5 (5,3 – 6,0)	0,952
CT (mg/dL)	174,5 ± 39,6	178,9 ± 38,0	171,8 ± 40,7	0,444
HDL (mg/dL)	49,5 ± 15,0	52,2 ±16,4	47,8 ±13,9	0,211
LDL (mg/dL)	99,5 ± 33,4	98,5 ± 29,2	100,1 ± 35,9	0,839
Triglicerídeos (mg/dL)	126,0 (98,5–170,5)	116,5 (93,8– 56,0)	131,0 (108,0-171,0)	0,162
OPG (pmol/L)	20,6 (17,1- 24,9)	23,8 (20,7-30,2)	18,1 (3,8-21,0)	**<0,001**

Teste t ou teste de Mann-Whitney; IA: índice de aumento; PAS: pressão arterial sistólica; PAD: pressão arterial diastólica; HG: hemoglobina glicada; VOP: velocidade de onda de pulso; CT: colesterol total; HDL: lipoproteína de alta densidade; LDL: lipoproteína de baixa densidade; OPG: osteoprotegerina; PPC: pressão de pulso central; DFM: dilatação fluxo-mediada; DR: disfunção renal.

Os valores de ureia e creatinina foram, conforme esperado, mais altos nos pacientes com DR. OPG foi mais alto no grupo com DR. Todos os demais testes laboratoriais foram similares entre os grupos (Tabela 4).

Os valores de OPG aumentaram com a gravidade da albuminúria, mas sem significância estatística. Pacientes com DRC estágio 3ª ou 3b apresentaram valores de OPG mais altos (Tabela 5).

**Tabela 5:** – Comparação dos níveis de osteoprotegerina de acordo com albuminúria e classificação da Taxa de Filtração Glomerular (TFG)

	OPG	p
**Albuminúria (mg/dL)**		0,126**
Estágio 1	19,8 (15,4- 23,7)	
Estágio 2	21,0 (18,6 – 30,6)	
Estágio 3	28,8 (21,9 – 29,7)	
**Classificação da disfunção rena (mL/min/1,73m^2^)**		< 0,001**
Estágio 1	18,8 (16,8-21,2)^a^	
Estágio 2	18,6 (15,7-20,7)^a^	
Estágio 3a	23,7 (20,9-30,7)^b^	
Estágio 3b	27,4 (21,8-29,3)^b^	
**Classificação da disfunção renal (ml/min/1,73m^2^)**		< 0,001*
Estágio 1 e 2	18,1 (13,8 – 21,0)	
Estágio 3a e 3b	23,8 (20,7 – 30,1)	

TFG: taxa de filtração glomerular; OPG: Osteoprotegerina. *Mann-Whitney; **Kruskal-Wallis com teste post-hoc de Dunn. Letras iguais: sem diferença estatística; letras diferentes: com diferença estatística

Observou-se uma correlação positiva dos níveis de OPG com valores de pressão arterial sistólica, PAC, VOP e Aix. Não se observou correlação entre os níveis séricos de OPG e DFM (Tabela 6) (Figura Central).

**Tabela 6:** – Correlação entre osteoprotegerina e outras variáveis

	Com DR		Sem DR	
	r	p	r	p
TFG (CKD-EPI) (ml/min/1,73 m^2^)	0,248	0,086	-0,135	0,478
**Pressão arterial**				
PAS periférica (mmHg)	-0,001	0,995	0,426	**0,019**
PAD periférica (mmHg)	-0,101	0,492	0,286	0,126
**Medida de Pressão Arterial Central**				
PAS central (mmHg)	0,005	0,972	0,409	**0,025**
PAD central (mmHg)	-0,053	0,719	0,080	0,675
PPC (mmHg)	0,159	0,275	0,496	**0,005**
VOP (m/sec)	-0,205	0,158	0,483	**0,007**
IA (%)	0,094	0,518	0,369	**0,045**
**DFM (%)**	-0,002	0,164	-0,010	0,959

Teste de correlação de Pearson ou de Spearman. CKD-EPI: Chronic Kidney Disease Epidemiology Collaboration; TFG: taxa de filtração glomerular; IA: índice de aumento; PAS: pressão arterial sistólica; PAD: pressão arterial diastólica; VOP: velocidade de onda de pulso; CT: colesterol total; OPG: Osteoprotegerina; PPC: pressão de pulso central; DFM: dilatação fluxo mediada; DR: disfunção renal.

## Discussão

O presente estudo avaliou diferentes parâmetros da dinâmica vascular em pacientes com DRC não dialítica e demonstrou alguns resultados importantes.

Primeiro, pacientes com DRC estágio 3 apresentaram valores mais altos de VOP, PPC, e IA em comparação aos controles, apesar de valores similares de pressão arterial entre os grupos. Rigidez arterial central é um dos melhores biomarcadores para estratificar o risco de DCV em paciente com doença renal em estágio terminal, principalmente aqueles que necessitam de diálise.^10,11,29^ Outros estudos também demonstraram rigidez arterial aumentada em pacientes com lesão renal incipiente.^30-32^ Portanto, rigidez arterial aumentada pode ser um fator importante na identificação do fenótipo de doença vascular na DRC.

Segundo, a OPG mostrou correlações fracas, mas significativas com os valores de pressão arterial periférica e central, e correlações moderadas com VOP, PPC e IA. Esses achados sugerem que uma rigidez arterial aumentada em pacientes com DRC estágio 3 (DR moderada) esteja relacionada à calcificação vascular na camada medial dos vasos. Estudos prévios demonstraram uma associação independente entre concentração sérica de OPG e o grau de rigidez vascular, medida por VOP em pacientes com vários estágios de DRC.^33-36^

No estudo KNOW-CKD, observou-se uma forte correlação entre VOP e idade. Além disso, a segunda variável com maior correlação com VOP foi a OPG em uma regressão multivariada de pacientes não dialíticos. Hyun et al. também encontraram uma correlação positiva entre os níveis de OPG e PPC. Ainda, os autores demonstraram que um aumento na OPG em 1 pmol/L levou a um aumento na VOP de 17,1 cm/segundo em média.^34^

No presente estudo, a OPG não se associou com albuminúria. No entanto, sabe-se que a albuminúria é um marcador de lesão endotelial, e estudos prévios demonstraram uma correlação entre OPG sérica e albuminúria, especialmente em pacientes com DM tipo 2.^37,38^ Tal correlação não foi detectada neste estudo, possivelmente devido ao nosso pequeno tamanho amostral e o pareamento de pacientes com DM, em que a albuminúria é mais frequente.

O incremento de cada 1pmol/L nos níveis séricos de OPG aumenta o risco de morte por DCV em 4% nos pacientes com DRC.^39^ Quanto mais alto o nível de OPG, maior a mortalidade por todas as causas em pacientes com DRC estágios 3-5.^40,41^Em nosso estudo caso-controle, não foi possível avaliar a associação direta da OPG com risco CV e mortalidade em pacientes com DRC. Contudo, a OPG é possivelmente um bom marcador precoce de disfunção vascular incipiente em pacientes com DRC moderada.

Terceiro, pacientes no grupo com DR não apresentaram valores mais altos de DFM (p=0,671) e não observamos uma correlação com OPG (0,959). A associação entre DFM e DRC foi mais prevalente nos pacientes com DRC avançada, DM e albuminúria, mesmo sem a presença de doença arterial coronariana estabelecida.^6,42,43^ Por outro lado, em pacientes com DRC em estágios menos avançados, os estudos são limitados e os resultados controversos.^5,7,43^No presente estudo, os valores de DFM foram similares entre os grupos, o que sugere que o dano endotelial pode intensificar somente em estágios mais avançados de DRC.

No estudo Hoorn,^7^ em que a avaliação da disfunção vascular em pacientes com DRC incipiente foi realizada por medida de biomarcadores laboratoriais – fator de Von Willebrand, molécula de adesão intercelular-1, e razão albumina/creatinina – observou-se uma correlação inversa desses marcadores com o grau de disfunção endotelial.^7^ Porém, essa mesma associação não foi repetida em outros estudos que avaliaram disfunção endotelial por meio de DFM em pacientes com DRC moderada.^7,31^ Vale mencionar que os marcadores avaliados no estudo Hoorn são também marcadores de hemostasia e trombose e, por isso, podem refletir esses processos e não a função endotelial.

No estudo de Iwamoto et al.,^43^ a disfunção endotelial foi encontrada em pacientes não dialíticos com DRC por vasodilatação induzida por nitroglicerina, que detecta aterosclerose pela avaliação da função do músculo liso vascular. Nossos resultados, em contraste, corroboram os resultados do *The Framingham Heart Study*, que também avaliou pacientes com DRC (estágio 3) e não mostrou correlação significativa positiva da disfunção endotelial (avaliada por DFM) com estágios menos avançados da DRC.^5^

Um estudo prévio mostrou que a associação da disfunção endotelial (medida pela DFM) em pacientes com DRC foi mais evidente em pacientes que não estavam tomando medicamentos anti-hipertensivos.^44^ Tal fato deve-se, provavelmente, a ações dos medicamentos, afetando a liberação de óxido nítrico e aumentando sua produção ou atividade, o que pode contribuir em um aumento de até dois pontos na porcentagem de DFM.^45^

A OPG é liberada principalmente por osteoblastos e células endoteliais. Essa molécula pode estar implicada na fisiopatologia da calcificação vascular e disfunção endotelial em pacientes com DRC.^46^ Importante mencionar que poucos estudos avaliaram a associação entre DFM e OPG em pacientes com DRC menos avançada. Yilmaz et al.^47^ encontraram uma associação independente entre OPG e DFM, mas somente 30,6% dos pacientes estavam em uso de IECA/BRA; 25% deles eram hipertensos. O estudo também incluiu pacientes com DRC em estágios mais avançados, diferentemente da população do nosso estudo.

A DFM pode não ser o método ideal para detectar alterações endoteliais precoces em pacientes com DRC. Ainda, a fisiopatologia das alterações endoteliais na DRC não é bem conhecida e pode ocorrer por diferentes vias, e não somente pela liberação de óxido nítrico. É possível que o alto risco CV encontrado nesses pacientes, mesmo nos estágios iniciais da DRC, esteja relacionado a outros mecanismos fisiopatológicos, tais como calcificação vascular, inflamação, e rigidez arterial. São necessários outros estudos prospectivos para melhor avaliar a relação entre DFM e os estágios da DRC.

Em resumo, os achados deste estudo podem estar diretamente relacionados à fisiopatologia da calcificação e à inflamação vascular na DRC incipiente. A calcificação da túnica íntima está mais relacionada à deposição de lipídios e à infiltração inflamatória, o que levaria à disfunção endotelial.^48^ Na túnica média, a calcificação está mais relacionada à transformação das células do músculo liso vascular em células similares a osteoblastos.^16^ É possível que, nesse estágio da DRC, a calcificação ocorra mais cedo na túnica média que na íntima vascular, o que explicaria os níveis mais altos de OPG e a rigidez arterial significativamente mais alta nesses pacientes. A doença aterosclerótica aumenta à medida que a TFG diminui. Além disso, mesmo nos pacientes com lesão renal incipiente, é possível que a disfunção endotelial e a inflamação exerçam um importante papel no desenvolvimento de doença aterosclerótica precoce.^7^

O estudo tem algumas limitações. Este é um estudo caso-controle conduzido em um único centro. O tamanho da amostra foi pequeno, com um número limitado de pacientes com DRC, representando uma população mais velha, mas com algumas diferenças em idade entre os grupos. O uso de anti-hipertensivos pode ter alterado a liberação de óxido nítrico e influenciado as medidas de DFM. Ainda, a disfunção endotelial foi avaliada usando somente um biomarcador, que pode não representar completamente a fisiopatologia da alteração endotelial na DRC. Outros estudos longitudinais são necessários para melhor compreender as correlações entre esses biomarcadores.

## Conclusão

Encontramos níveis mais elevados de VOP, PPC, e IA e uma correlação moderada da OPG com VOP, PPC e IA em paciente com DRC estágio 3. Esses dados reforçam que a disfunção vascular já pode estar presente em pacientes com DR em estágios moderados.
