# Supplementary material for: Osteoprotegerina e Disfunção Vascular em Pacientes com Doença Renal Crônica Estágio 3 e em Pacientes sem Disfunção Renal: Estudo Caso-Controle
Source: Arq Bras Cardiol. 2024 Nov 7;121(11):e20240122. [Article in Portuguese] doi: 10.36660/abc.20240122 (PMC11634311; doi:10.36660/abc.20240122)
Supplement: Translation

# Translation: Osteoprotegerin and Vascular Dysfunction in Patients with Stage 3 Chronic Kidney Disease and Those without Renal Dysfunction: A Case-Control Study

**DOI:** 10.36660/abc.20240122i

**Authors:** Thalita de Oliveira Matos, Priscila Valverde de Oliveira Vitorino, Rogerio Orlow, Átila de Oliveira Melo, Diogo da Silva Amorim, Gleidson Junio Oliveira Sousa, Vanda Jorgetti, Ana Luiza Lima Sousa, Rodrigo Bezerra, Weimar Kunz Sebba Barroso

**Affiliations:** 1 Brazil Universidade Federal de Goiás – Liga de Hipertensão, Goiânia, GO – Brazil; 2 Brazil Pontifícia Universidade Católica de Goiás – Escola de Ciências Sociais e da Saúde, Goiânia, GO – Brazil; 3 Brazil Universidade Federal de Goiás, Goiânia, GO – Brazil; 4 Brazil Universidade de São Paulo, São Paulo, SP – Brazil; 5 Brazil Universidade de Pernambuco, Recife, PE – Brazil

**Keywords:** Vascular Stiffness, Vascular Calcification, Osteoprotegerin, Vascular Endothelium, Cardiovascular Diseases, Chronic Renal Insufficiency

## Abstract

**Background:**

Osteoprotegerin (OPG) is a marker of vascular calcification and cardiovascular risk in patients with chronic kidney disease (CKD).

**Objective:**

This study aimed to compare and correlate OPG values with flow-mediated dilation (FMD) and pulse wave velocity (PWV) measurements in patients in stage 3 CKD and those without renal dysfunction.

**Methods:**

This case-control study was conducted in a specialized hypertension center in 2022. A total of 79 patients over 18 years of age participated in the study. The case group consisted of 30 patients with moderate renal dysfunction (CKD stage 3) and the control group included 49 individuals with glomerular filtration rate ≥ 60 mL/min/1.73 m^2^. The significance level adopted in the statistical analysis was 5%.

**Results:**

Central pulse pressure (cPP), PWV, and augmentation index (AIx) were higher in patients with renal dysfunction. The serum OPG level positively correlated with peripheral and central systolic blood pressure, cPP, PWV, and AIx. Conversely, the serum OPG did not correlate with FMD.

**Conclusions:**

OPG and PWV are possible biomarkers of vascular dysfunction that are altered in patients with moderate renal dysfunction. Despite limitations of this study, including that it was a case-control study conducted at a single center, it has the potential, as a proof of concept, to generate the hypothesis of OPG and PWV as biomarkers of early vascular damage in this population.

## Introduction

Patients with chronic kidney disease (CKD) are at an increased risk for cardiovascular disease (CVD) due to traditional risk factors (RF), such as diabetes mellitus (DM), hypertension (HT), dyslipidemia (DLP), smoking, and age. This risk is also enhanced by non-traditional RF, such as low bone and mineral disorder, chronic inflammation, anemia, increased uremic toxins, endothelial nitric oxide depletion, accumulation of glycosylation end products, over-reactivation of the renin-angiotensin-aldosterone system (RAAS), and oxidative stress. These RF potentiate the increased endothelial dysfunction, vascular calcification and osteogenesis found in patients with CKD.^1,2^

Endothelial dysfunction is associated with the development of CVD, and flow-mediated dilation (FMD) is the method used for its assessment. Although FMD is regarded as the gold standard,^3,4^ findings from studies in patients with CKD remain controversial, especially in those with less advanced stages of the disease.^5-7^

Another method used to identify early vascular damage is central blood pressure measurements (cBPM) and pulse wave velocity (PWV) analysis. Both measurements are good surrogate endpoints^8^ and independent predictors of CVD mortality in those with CKD.^9-11^ Every 1m/sec increase in PWV corresponds to a 15%-30% increase in the risk of cardiovascular (CV) death.^12^Moreover, an increase in central pulse pressure (cPP) to levels greater than 50 mmHg is associated with worsening of renal function and adverse CV outcomes.^12-14^

Vascular calcification is commonly found in patients with advanced-stage CKD.^15^ These patients present phenotypic changes that culminate in homeostatic disturbances of calcium, phosphorus, parathormone, and calcitriol, in addition to disorders related to bone metabolism and remodeling, with increased resorption and heterotopic calcification, especially vascular calcification.^16,17^

Vascular calcification is an active process in which there are alterations in the vessel matrix composition and phenotypic changes from smooth muscle cells to *osteoblast-like* cells, as well as the presence of osteogenesis modulating proteins and an imbalance between the stimulating and inhibiting factors of vascular calcification.^17-19^

Osteoprotegerin (OPG) is a glycoprotein of the tumor necrosis factor (TNF) superfamily that prevents the binding of the RANK-L to the receptor activator of nuclear factor kappa B (RANK), thereby inhibiting osteoclast activation. OPG is, therefore, believed to inhibit osteoclastogenesis, protect bone volume and bone mass, and inhibit vascular calcification.^20^ Circulating levels of OPG are elevated in patients who require dialysis and may be a strong predictor of vascular calcification at various stages of kidney disease.^21-23^

Vascular damage is possibly one of the determinants of high CV risk in patients with CKD. The detection of this damage in the early stages of the disease may contribute to the adoption of strategies with the potential to reduce CV and renal morbidity and mortality. This study aimed to verify the correlation of serum OPG with different degrees of vascular dysfunction in patients with CKD stages 3a and 3b versus those without renal dysfunction.

## Methods

This is a case-control study that included patients from a hypertension referral center – Arterial Hypertension League at the Federal University of Goiás. The study included 79 participants with one or more CV RF. The participants were classified according to their renal function as determined by the glomerular filtration rate (GFR) estimated by the CKD epidemiology collaboration equation (2021). In this study, patients with a GFR <60 mL/min/1.73 m^2^ and ≥30 mL/min/1.73 m^2^ were classified as patients with CKD 3a and 3b, respectively. Subjects with CKD stage 3a have a GFR between 45 and 59 and those with stage 3b have a GFR between 30 and 44 mL/min/1.73 m^2^. Those with a GFR of ≥60 mL/min/1.73 m^2^ were classified as patients without renal dysfunction. The study was approved by the ethics committee of the Federal University of Goiás (FUG) general hospital (CAAE: 47327421.7.0000.5078).

Adults aged 18 years and older, with HT and with GFR ≥30 ml/min/1.73m^2^during regular follow-up in a HT referral center were included. The exclusion criteria included patients with CKD who required hemodialysis or peritoneal dialysis, patients with previous kidney transplantation; pregnant or postpartum women; patients diagnosed with cancer, those undergoing chemotherapy or radiotherapy, patients with a history of systemic autoimmune disease and on immunosuppressants, patients with advanced chronic hepatopathy (Child B and C), and those with previous CVD.

Prior CVD was defined as the presence of any of the following diagnoses: coronary disease (angina, acute myocardial infarction), heart failure, valvular heart disease, complex arrhythmias, obstructive peripheral arterial disease, aortic aneurysm, stroke, and atherosclerotic disease characterized by critical stenosis (>50%) in vascular beds. The diagnoses of HT, DM, and DLP were made based on the medical history recorded in the patient medical records and by the chronic and regular use of medications for the treatment of these diseases.

The groups of patients with or without renal dysfunction (RD) were defined following tests performed by a single laboratory. All patients with RD were recruited from the specialized referral center, and a control group of patients without RD (no RD), paired for the presence of DM and obesity was also included.

Participants underwent physical examinations and laboratory testing. Peripheral blood pressure (BP) was measured according to the technique recommended by the Brazilian Guidelines on Hypertension^24^ with an automated oscillometric device (Omron HBP-1100). Three consecutive measurements were taken with the patient in a sitting position, and the average of the last two measurements was recorded as the peripheral BP. At the same visit, blood was collected for biochemical tests and the cBPM and the FMD were also assessed (Table 1).

**Table 1:** – Tests used in the study for the assessment of risk factors for vascular dysfunction

Exam	Risk factor assessed
Osteoprotegerin	Vascular calcification
Flow-mediated dilatation, albuminuria	Endothelial function
Central blood pressure measurement/pulse wave velocity	Vascular rigidity

cBPM was performed using the standardized approach with the Dyna-MAPA-AOP device (Cardios, Brazil) by the oscillometric method. The method was applied according to the recommendations of the American Heart Association, 2015.^8^ The reference value for PWV for target organ damage was ≥10 m/s.

Laboratory testing included fasting glucose, glycated hemoglobin, lipid profile (total cholesterol and fractions), creatinine, urea, and albuminuria. Peripheral venous blood was drawn (approximately 30 mL) from the antecubital vein between 7 am and 9 am. All participants were required to fast for 12 hours. In addition, a 50 mL midstream urine sample was collected to evaluate for albuminuria.

Laboratory OPG evaluation was performed by collecting another 15 mL of blood. Each sample was centrifuged, and the supernatant was divided into two vials (2 mL Eppendorf) that were labeled and stored at -30^o^C. Samples that were hemolyzed or lipemic were discarded and recollected. Freeze/thaw cycles, which could cause erroneous results, were avoided.

Serum OPG level was determined using the sandwich ELISA technique (BioVendor, Czech Republic) following the manufacturer’s instructions. The essay uses an anti-OPG monoclonal antibody adhered to the plate to capture OPG in the serum. The captured OPG was detected by adding a second biotin-conjugated anti-OPG polyclonal antibody.

The evaluation of endothelial function by means of FMD was performed by a single trained examiner using the technique described by Celermajer et al. with a high-resolution ultrasound (US) device.^25^ The UNEX EF38G was used in the study, which has a robotic device that scans through the brachial artery in the patient’s arm automatically and uniformly, providing a more accurate assessment. It is currently the gold standard. The test was performed by inflating the cuff on the forearm to a pressure of 50 mmHg above systolic BP and maintained inflated for five minutes. Peak brachial artery diameter was measured by continuous, dynamic ultrasound assessment up to 180 seconds after deflation of the cuff.^4^ The risk for CVD is higher when the FMD <10%. In this study, the values higher than 10% were considered normal.^26^

To perform the cBPM and FMD, the patients were instructed to have a good night’s sleep the day before the exam, avoid drinks with caffeine 12 hours before the exam; avoid alcoholic beverages 12 hours before the exam, avoid smoking 6 hours before the exam, avoid physical exercises in the last 24 hours before the exam, rest for 20 minutes before the exam, continue existing medications, and fasting for at least 12 hours.^4,26^ Chart 1 summarizes the tests used in this study and the RF evaluated

### Statistical analysis

An electronic form was constructed using REDCap^27^ and extracted for statistical analysis with JAMOVI, version 2.2.^28^ The Shapiro-Wilk test was used to verify the data distribution of the variables. Descriptive analysis was expressed with absolute and relative frequency for qualitative variables and with mean ± standard deviation or median and interquartile range for quantitative variables, according to the normality of the data. The significance level adopted in the statistical analysis was 5%. To constitute the sample, a sample calculation was carried out.

For a mean difference of 4.4 ± 2.1 pmol/L in OPG between patients in the first (4.9 ± 1.9 pmol/L) and fourth tertile (9.3 ± 4.0 pmol/L) a sample of 19 participants was obtained in each of the groups. Due to the risk of losing samples collected for OPG analysis or difficulties in performing FMD on a patient, we chose to increase our sample size to ensure that the study provides a meaningful sample.

For the comparative analysis between patients with and without RD, the chi-square test was used for qualitative variables and the independent t-test or Mann-Whitney test was used for quantitative variables. The comparative analysis of OPG according to the Kidney Disease: Improving Global Outcomes (KDIGO) guideline classification of CKD was done using Kruskal-Wallis with Dunn’s post-hoc test between patients of different CKD stages. Correlations of OPG with the other variables were analyzed using Pearson’s or Spearman’s correlation tests.

## Results

Mean age of participants was 64.1±9.7 years. Patients with RD were older – 70.1±7.2 vs. 60.0±8.7 years (p <0.001) and had lower GFR values. Sex distribution between the groups was similar (Table 2). The RD group was composed of 30 patients and the NRD group was composed of 49 patients.

**Table 2:** – Sample description and comparison between patients with and without renal dysfunction

	Total	With dysfunction	No dysfunction	p
Age (years)	64.1 ± 9.7	70.1 ± 7.2	60.0 ± 8.7	**< 0.001**
Body mass index (kg/m^2^)	29.7 ± 5.9	28.9 ± 5.13	30.3 ± 6.38	0.301
GFR (mL/min/1.73 m^2^)	70.0 (50.5-88.0)	47.0 (42.3-53.0)	85.0 (72.0 – 95.0)	**<0.001**
≥ 65 years old	40 (50.6%)	22 (73.3%)	18 (36.7%)	**0.002**
Female	47.0 (59.5 %)	18 (60.0%)	29 (59.2%)	1.000
Diabetes mellitus	27 (34.2%)	12 (40.0%)	15 (30.6%)	0.542
Dyslipidemia	60 (78.9%)	23 (76.7%)	37 (80.4%)	0.916

Independent t-test or Mann-Whitney or chi-square test. GFR: glomerular filtration rate.

The use of combination antihypertensives was evidenced in 82.3% of the sample. Angiotensin-converting enzyme inhibitors (ACEI) and angiotensin receptor blockers (ARB) were the most frequently used. Of the patients who were on diuretics, none was on loop diuretics. The diuretics used were thiazide, thiazide-like, and potassium sparing diuretics (Table 3).

**Table 3:** – Frequency, number of antihypertensive drugs, and antihypertensive classes used by all study participants, and by patients with and without renal dysfunction

	Total (79 patients) n (%)	With dysfunction (30 patients) n (%)	No dysfunction (49 patients) n (%)
**Number of antihypertensives**			
One	14 (17.7)	02 (6.6)	12 (24.4)
Two	24 (30.4)	10 (33.3)	14 (28.5)
Three	24 (30.4)	12 (40.0)	12 (24.4)
More than three	17 (21.5)	06 (20.0)	11 (22.4)
**Classes of antihypertensives**			
ACEI/ARB	74 (93.7)	28 (93.3)	46 (93.8)
Diuretics	55 (69.6)	20 (66.6)	35 (73.4)
CCB	42 (53.2)	20 (66.6)	22 (44.9)
Vasodilators	06 (7.6)	02 (6.6)	04 (66.7)
b-blockers	21 (26.6)	10 (33.3)	11 (52.4)

ARB: angiotensin receptor blockers; ACEI: Angiotensin-converting enzyme inhibitors; CCB: calcium channel blocker.

The systolic and diastolic BP values, both by peripheral and central measurements, were similar between the groups. No differences in FMD values were observed between the groups. Furthermore, cPP, PWV, and augmentation index (Aix) were higher in those in the RD group (Table 4).

**Table 4:** – Comparison of biochemistry results, peripheral blood pressure, central blood pressure measurement and DFM between those with and without renal dysfunction

Exams	Total	With RD	No RD	p
Peripheral SBP (mmHg)	137.0 (126.3–59.0)	146.3 (128.6–62.8)	135.5 (126.0–55.0)	0.208
Peripheral DBP (mmHg)	81.8±14.8	79.0±11.1	82.2±15.9	0.473
Central SBP (mmHg)	112.0 (103.0-127.5)	112.5 (105.2-136.0)	112.0 (103.0-127.0)	0.410
Central DBP	79.0 (70.0 – 91.5)	79.5 (69.3 – 90.3)	78.0 (71.0 – 93.0)	0.743
cPP (mmHg)	34.0 (29.0 – 40.0)	38.5 (32.0-47.0)	33.0 (27.5 – 37.0)	**0.006**
PWV (m/sec)	9.0 (8.6 – 10.2)	10.2 (9.2 – 11.9)	8.5 (7.7 – 9.5)	**<0.001**
Aix (%)	24.0 (16.0 – 31.5)	27.0 (21.1 – 34.0)	21.0 (14.5 – 29.0)	**0.012**
FMD (%)	8.7 (5.3 – 12.4)	9.2 (5.0 – 11.4)	8.4 (5.4 – 13.4)	0.671
Creatinine (mg/dL)	1.1 (0.9 – 1.3)	1.4 (1.2 – 1.6)	0.9 (0.8 – 1.0)	**<0.001**
Urea (mg/dL)	37.0 (29.0 – 45.0)	46.6 (39.0 – 58.8)	32.0 (25.5 – 71.5)	**<0.001**
Albuminuria (mg/g)	12.0 (5.0 – 28.2)	9.2 (2.0 – 22.8)	17.5 (7.6 – 61.4)	0.065
Blood glucose (mg/dL)	95.0 (87.5 – 105.5)	95.0 (86.0 – 100.0)	97.0 (92.0 – 109.0)	0.168
GH (%)	5.7 (5.4 – 6.05)	5.8 (5.5 – 6.3)	5.5 (5.3 – 6.0)	0.952
TC (mg/dL)	174.5 ± 39.6	178.9 ± 38.0	171.8 ± 40.7	0.444
HDL (mg/dL)	49.5 ± 15.0	52.2 ±16.4	47.8 ±13.9	0.211
LDL (mg/dL)	99.5 ± 33.4	98.5 ± 29.2	100.1 ± 35.9	0.839
Triglycerides (mg/dL)	126.0 (98.5–170.5)	116.5 (93.8– 56.0)	131.0 (108.0-171.0)	0.162
OPG (pmol/L)	20.6 (17.1- 24.9)	23.8 (20.7-30.2)	18.1 (3.8-21.0)	**<0.001**

t-test or Mann-Whitney test. Aix: augmentation index; FMD: measured flow dilation; BP: blood pressure; DBP: diastolic blood pressure; SBP: systolic blood pressure; cPP: central pulse pressure; GH: Glycated hemoglobin; PWV: pulse wave velocity; TC: total cholesterol; HDL: high density lipoprotein; LDL: low density lipoprotein; OPG: osteoprotegerin; RD: renal dysfunction.

The values of urea and creatinine were, as expected, higher in the RD group. OPG was higher in the RD group. All other laboratory tests were similar between the groups (Table 4).

The OPG values increased with albuminuria severity, although without statistical significance. Patients with stage 3a or 3b CKD had higher OPG values (Table 5).

**Table 5:** – Comparison of the osteoprotegerin level according to the albuminuria and GFR classifications

	OPG	p
**Albuminuria (mg/dL)**		0.126**
Stage 1	19.8 (15.4- 23.7)	
Stage 2	21.0 (18.6 – 30.6)	
Stage 3	28.8 (21.9 – 29.7)	
**Classification of renal dysfunction (ml/min/1.73m^2^)**		< 0.001**
Stage 1	18.8 (16.8-21.2)^a^	
Stage 2	18.6 (15.7-20.7)^a^	
Stage 3a	23.7 (20.9-30.7)^b^	
Stage 3b	27.4 (21.8-29.3)^b^	
**Classification of renal dysfunction (ml/min/1.73m^2^)**		< 0.001*
Stage 1 and 2	18.1 (13.8 – 21.0)	
Stage 3a and 3b	23.8 (20.7 – 30.1)	

GFR: glomerular filtration rate; OPG: osteoprotegerin. *Mann-Whitney; **Kruskal-Wallis with Dunn posthoc test. Same letters - no statistical difference and different letters – with statistical difference.

There was a positive correlation of serum OPG levels with peripheral and central systolic BP, cPP, PWV, and Aix values. No correlation was found between the serum OPG level and FMD (Table 6) (Central Illustration).

**Table 6:** – The correlation between osteoprotegerin and other variables

	No renal dysfunction		With renal dysfunction	
	r	p	r	p
GFR (CKD-EPI) (ml/min/1.73 m^2^)	0.248	0.086	-0.135	0.478
**Blood pressure**				
Peripheral SBP (mmHg)	-0.001	0.995	0.426	**0.019**
Peripheral DBP (mmHg)	-0.101	0.492	0.286	0.126
**Central blood pressure measurement**				
Central SBP (mmHg)	0.005	0.972	0.409	**0.025**
Central DBP (mmHg)	-0.053	0.719	0.080	0.675
cPP (mmHg)	0.159	0.275	0.496	**0.005**
PWV (m/sec)	-0.205	0.158	0.483	**0.007**
Aix (%)	0.094	0.518	0.369	**0.045**
**FMD (%)**	-0.002	0.164	-0.010	0.959

Pearson’s or Spearman’s correlation test. Aix: index augmentation. CKD-EPI: Chronic Kidney Disease Epidemiology Collaboration; FMD: flow-mediated dilation; PWV: pulse wave velocity; DBP: diastolic blood pressure; SBP: systolic blood pressure; GFR: glomerular filtration rate; cPP: central pulse pressure.

## Discussion

The present study evaluated different parameters of vascular dynamics in patients with non-dialytic CKD and demonstrated some important results.

First, patients in stage 3 CKD had higher values of PWV, cPP, and Aix compared to the controls, despite similar BP values between the groups. Central arterial stiffness is one of the best biomarkers to stratify the risk of CVD in patients with end stage renal disease (ESRD), especially those who require dialysis.^10,11,29^ Other studies have also demonstrated increased arterial stiffness even in patients with incipient kidney damage.^31-32^ Therefore, increased arterial stiffness may be an important factor in identifying the phenotype of vascular disease in CKD.

Second, OPG showed weak but significant correlations with peripheral and central BP values, and moderate correlations with PWV, cPP, and AIx. These findings suggest that increased arterial stiffness in patients with stage 3 CKD (moderate RD) is related to early vascular calcification in the middle layer of the vessels. Previous studies demonstrated an independent association between serum OPG and the degree of vascular stiffness, measured by PWV in patients with various stages of CKD.^33-36^

In the KNOW-CKD study, a strong correlation was found between PWV and age. Moreover, the second most correlated variable with OPV was OPG in a multivariate regression of non-dialysis patients. Hyun et al. also found a positive correlation between OPG and cPP levels. Furthermore, they demonstrated that an increase in OPG by 1 pmol/L, led to a raise in PWV by an average of approximately 17.1 cm/second.^34^

OPG was not associated with albuminuria in the present study. However, it is known that albuminuria is a marker of endothelial injury and previous studies have demonstrated a correlation between serum OPG and albuminuria, especially in patients with DM2.^37,38^ This association was not detected in this study, possibly because of our small sample size and the pairing of patients with DM, in which the finding of albuminuria is more frequent.

Every 1 pmol/L increase in serum OPG increases the risk of CV death by 4% in patients with CKD.^39^The higher the OPG level, the higher the all-cause mortality in patients with CKD stages 3-5.^40,41^In our case-control study, it was not possible to assess the direct association of OPG with CV risk and mortality. However, this is possibly a good early marker of incipient vascular dysfunction in patients with moderate CKD.

Third, patients in the RD group did not have higher FMD values (p =0.671) and a correlation with OPG was not detected (p =0.959). The association between FMD and CKD was more prevalent in patients with advanced CKD, DM, and albuminuria, even without the presence of established coronary artery disease.^6,42,43^ In contrast, in patients with CKD in less advanced stages, studies are limited and the results are controversial.^5,7,43^In the current study, the FMD values were similar between the groups, which suggests that endothelial damage may intensify only in more advanced stages of CKD.

In the Hoorn study,^7^ in which the assessment of vascular dysfunction in patients with incipient CKD was performed through the measurement of laboratory biomarkers: Von Willebrand factor, intercellular adhesion molecule-1, and albumin/creatinine ratio, there was an inverse correlation of these markers, and the degree of endothelial dysfunction.^7^However, this same association was not repeated in other studies that evaluated endothelial dysfunction by means of FMD in patients with moderate CKD.^7,31^Notably, the biomarkers evaluated in the Hoorn study are also markers of hemostasis and thrombosis, and thus, may reflect these processes other than endothelial function.

In the study by Iwamoto et al.,^43^ endothelial dysfunction was found in non-dialytic patients with CKD by means of nitroglycerin-induced vasodilation, which detects atherosclerosis by assessing the vascular smooth muscle function. Our findings, in contrast, corroborate with the findings of The Framingham Heart Study, which also evaluated patients with CKD (stage 3) and showed no significant positive correlation in endothelial dysfunction (assessed by FMD) with less advanced stages of CKD.^5^

A previous study showed that the association of endothelial dysfunction (measured by the FMD) in patients with CKD was more evident in patients who were not taking antihypertensive drugs.^44^ This is possibly due to the drug actions, affecting the release of nitric oxide and increasing its production or activity, which may contribute to an increase of up to two points in the % FMD value.^45^

OPG is released mainly by osteoblasts and endothelial cells. This molecule may be implicated in the pathophysiology of vascular calcification and endothelial dysfunction in patients with CKD.^46^Importantly, few studies have evaluated the association between FMD and OPG in patients with less advanced CKD. Yilmaz et al.^47^ found an independent association between OPG and FMD, but only 30.6% of patients were on ACEI/ARB; 25% of patients were hypertensive. The study also included patients in more advanced stages of CKD, differently from our study population.

FMD may not be an ideal way for detecting very early endothelial changes in patients with CKD. In addition, the pathophysiology of endothelial changes in CKD is not well known and may occur through several pathways, and not only through the release of nitric oxide. It is also possible that the high CV risk found in these patients even in the early stages of CKD may be related to other pathophysiological mechanisms, such as vascular calcification, inflammation, and arterial stiffness. Further prospective studies to better evaluate the relationship between FMD and various stages of CKD are warranted.

In summary, the findings of this study may be directly related to the pathophysiology of calcification and vascular inflammation in incipient CKD. Intima tunica calcification is more related to lipid deposition and inflammatory infiltration, which would lead to endothelial dysfunction.^48^In the tunica media, calcification is more related to the transformation of the vascular smooth muscle cells into *osteoblastic-like* cells.^16^ It is possible that in this stage of CKD, calcification occurs earlier in the tunica media than in the vascular intima, which would explain the higher OPG levels and the significantly higher arterial stiffness among these patients. Atherosclerotic disease increases as the GFR reduces. Also, even in patients with incipient renal damage, endothelial dysfunction, and inflammation may play an important role in the development of early atherosclerotic disease.^7^

The study has some limitations. It is a case-control study conducted in a single center. The sample size was small with a limited number of patients with CKD, representing an older population, but with some differences in age between the groups. The use of antihypertensive medications may have altered the endothelial nitric oxide release and influenced the FMD measurements. Moreover, endothelial dysfunction was assessed using only one biomarker, which may not fully represent the pathophysiology underlying the endothelial alteration in CKD. More longitudinal studies are needed to better understand the correlations among these biomarkers.

## Conclusion

We found higher PWV, cPP, and Aix values and a moderate correlation of OPG with PWV, cPP, and AIx among patients with stage 3 CKD. These data reinforce that vascular dysfunction may be already present in patients with moderate stages of RD.
